# Debris Extrusion Using Reciproc Blue and XP Endo Shaper Systems in Root Canal Retreatment

**DOI:** 10.1155/2021/6697587

**Published:** 2021-03-24

**Authors:** Taher AlOmari, Ruba Mustafa, Rami Al-Fodeh, Hamza El-Farraj, Waheeb Khaled, Ahmed Jamleh

**Affiliations:** ^1^Department of Conservative Dentistry, Faculty of Dentistry, Jordan University of Science and Technology, Irbid, Jordan; ^2^Department of Prosthetic Dentistry, Faculty of Dentistry, Jordan University of Science and Technology, Irbid, Jordan; ^3^Department of Restorative and Prosthetic Sciences, College of Dentistry, King Saud Bin Abdulaziz University for Health Sciences, Riyadh, Saudi Arabia; ^4^King Abdullah International Medical Research Center, Ministry of National Guard-Health Affairs, Riyadh, Saudi Arabia

## Abstract

This study aimed at assessing the performance of Reciproc Blue (RB) and XP Endo Shaper (XPS), used for canal retreatment on extracted teeth, in terms of debris extrusion and obturating materials removal. Thirty mandibular premolars were prepared to ProTaper X2 file, obturated using warm vertical compaction, and then incubated for 28 days at 100% humidity at 37°C. Teeth were randomly assigned into two groups, according to the system used for retreatment (RB and XPS). During retreatment, debris extruded beyond the apex was collected in preweighed Eppendorf tubes, and the retreatment time was measured. Afterward, the teeth were longitudinally sectioned to assess the remaining obturating materials. Data were statistically analyzed using the Mann–Whitney test and chi-square test at a 95% confidence level. All the samples had extruded debris at varying weights ranging from 0.125 mg to 3.680 mg. XPS extruded less debris than RB, but no difference was detected (Mann–Whitney test; *P* > 0.05). RB and XPS required 54.9 ± 17.9 and 22.3 ± 9.3 seconds to perform retreatment procedures, respectively (Mann–Whitney test; *P* < 0.05). The sealer was found in all the samples. Compared to the RB group, fewer samples with remaining gutta-percha were found in the XPS group (Chi-square test; *P* < 0.05). None of the files fractured during the retreatment procedure. The tested files appear to extrude debris beyond the apex. Although XPS was able to remove the gutta-percha completely from the majority of the canals, it was unable to remove the sealer.

## 1. Introduction

The endodontic procedure aims at decreasing pain, eliminating disease, and sustaining or establishing healthy periapical tissues. This is achieved by using endodontic nickel-titanium (NiTi) rotary files to remove necrotic tissue and facilitate irrigation and medicament placement. In the case of failed treatment, nonsurgical root canal retreatment is required [1]. This procedure is initially performed by removal of the obturating material to regain access to the apical tissues [2]. However, removing the obturating filling completely is not yet possible with the available techniques [3–5], and this necessitates the search for improved techniques.

One of the challenges encountered during root canal procedures is debris extrusion, which is associated with an increased incidence of postoperative pain [6–8]. Previous studies have shown that root canal preparation with various rotary systems causes debris extrusion [9–16].

A single file concept that can be operated with different motions was introduced in endodontic practice to facilitate treatment without compromising the outcome [17]. According to a systematic review, file design and motion kinematics influence the amount of extruded debris more significantly than the number of files used [18]. This raised the concern of whether reciprocation can push out more debris than continuous rotation [19].

Reciproc Blue (RB; VDW, Munich, Germany) is a single file system designed to be used with reciprocating motion. It is manufactured from an M-wire alloy with special heat treatment. This technology shows increased file flexibility and improved cyclic fatigue resistance [20]. RB file is intended for use in initial treatment and retreatment [14, 16].

XP Endo Shaper (XPS; FKG Dentaire SA, La Chaux-de-Fonds, Switzerland) is a system which is manufactured from Max Wire. It has a snake-like shaped file to maximize the efficacy of cleaning the root canal system with the ability to touch most of the canal's walls. This system can reportedly be used in initial treatment and retreatment [12].

Since investigating the debris extrusion beyond the apex would provide pertinent information on file performance in a clinic, several studies have analyzed debris extrusion by several NiTi rotary endodontic files clinically used [12, 14, 16]. Thus, this in vitro study aimed to assess the performance of RB and XPS for root canal retreatment on extracted teeth in terms of debris extrusion and obturating materials removal.

## 2. Materials and Methods

### 2.1. Teeth Selection

This research was approved by the Institutional Research Ethics Committee (Ref. No. IRB/23/2017).

Single-canalled mandibular premolar teeth that had been extracted for orthodontic reasons were selected for the study. Periapical radiographs were taken in the mesiodistal and buccolingual planes, and the teeth were evaluated in order to exclude teeth with external defects, canal curvature larger than 10°, open apices, or other anatomic irregularities. The superficial tissues on the roots were mechanically removed using a scaler, and the teeth were stored in a thymol solution.

The sample size was calculated using a two-sample *t*-test at a power of 80% and 5% significance level to detect a minimum debris extrusion weight of 0.3 mg between the experimental groups. The results indicated that each group should be composed of 15 teeth.

### 2.2. Sample Preparation

The occlusal reduction was performed to obtain a reproducible reference point and to ensure the standardization of a 16 mm root length. An access cavity was performed, and patency was checked with K-type hand files. Any canal with an initial file size larger than size 25 was excluded.

### 2.3. Canal Preparation and Obturation

The working length was determined 1 mm short of the tooth length. The canals were instrumented with ProTaper Next X1 (17/04) and X2 (25/06) files (Dentsply Sirona, Ballaigues, Switzerland) and then obturated with gutta-percha cones and AH Plus sealer (Dentsply De Trey, Konstanz, Germany) using warm vertical compaction. The quality of the root canal obturation was confirmed radiographically, and any obturation deemed substandard was replaced with another sample. The access cavity was filled with Cavit^TM^ (3M ESPE, St Paul, MN, USA), and all samples were kept in phosphate-buffered saline with 100% humidity for 28 days at 37°C.

### 2.4. Retreatment Procedure

The temporary filling was removed, and the coronal third of the obturating material was removed with Gates-Glidden drills of sizes 3 and 4. Subsequently, the retreatment procedure was carried out with the experimental files that were operated as follows:

1. The RB group: R25 file (25/08) was operated in the “Reciproc All” mode using the VDW Silver Reciproc electric motor (VDW, Munich, Germany)
2. The XPS group: XPS file (27/01) was operated at a speed of 3000 rpm and 1 N·cm torque [12] using the Elements motor (SybronEndo, Glendora, CA)

The file was used with a slow pecking motion to remove the obturating materials. Once the working length was attained, the file was used for twenty brushing strokes along the whole canal length. Then, the canal was dried by canal aspiration and clinically explored through the access opening for any obturating material that might have remained on the wall. If any obturation material was left, another twenty brushing strokes were applied.

A single endodontist conducted all of the retreatment procedures using 4X magnifying loupes. Throughout the retreatment procedure, the canal was kept wet with tridistilled water.

### 2.5. Evaluation of Debris Extrusion

The setting of debris collection was adopted from a previous study [12]. To give a summary, Eppendorf tubes were used to collect debris extruded during the retreatment procedure. The tubes were individually weighed five times with a microbalance with four decimals in grams (Citizen CX 220 Analytical Lab Balance, Internal Cal. Weighing Hook, USA), and their mean was calculated. Each tooth was placed inside a preweighed Eppendorf tube at the level of the cementoenamel junction and fixed by using a rubber stopper made of silicone impression material. This assembly was inserted into a glass ampoule to avoid any possible contamination of the Eppendorf tube during the retreatment procedure.

Before initiating the retreatment procedure, a 27G irrigating needle was placed into the rubber stopper to equalize the inner and outer air pressure. The glass ampoule was firmly attached to the base of a larger outer glass container, which was submerged in a waterbath at a controlled temperature of 37°C, as confirmed with an electrode thermometer (MN35, Digital Mini MultiMeter, Boston, Massachusetts, USA).

During the retreatment, each tooth received a total of 3 ml of tridistilled water under close high vacuum suction. Finally, the canal was checked for patency with K-file size 15 and irrigated with another 2 ml of tridistilled water. The external surface of the apical third was also rinsed with 1 ml of tridistilled water to flush the apical surface and ensure the collection of any attached apical debris into the tube. The tooth was taken out, and the Eppendorf tube was moved to an incubator and kept for 2 weeks at 37°C to dry out the irrigating solution. Afterward, five weight measurements were obtained, and the mean value was calculated. The weight of extruded debris was calculated by measuring the difference between pre and postweights in mg. The total time needed for the preparation was recorded in seconds by a digital watch.

### 2.6. Evaluation of Gutta-Percha Removal

Each tooth was sectioned longitudinally and then split into two halves in the buccolingual aspect. The canals were evaluated under a dental operating microscope at 10X magnification (Zeiss OPMI Pico, Carl Zeiss Meditec AG, Germany) to assess the removal of sealer and gutta-percha.

### 2.7. Statistical Analysis

The Mann–Whitney test was performed to compare between the two groups since data of both weights of extruded debris and preparation time were not normally distributed (Shapiro–Wilk test; *P* < 0.05). The presence/absence of remaining gutta-percha in both groups was compared using the chi-square test. IBM SPSS 21 (SPSS Inc, Chicago, IL, USA) was used to perform statistical analyses with a 95% confidence level.

## 3. Results

The results of the apical extrusion of debris and removal of sealer and gutta-percha are given in Table 1. All the samples extruded debris at varying weights ranging from 0.125 mg to 3.680 mg. XPS extruded less debris than RB with a median of 1.145 mg (CI: 0.8471–1.8122) versus 1.235 mg (CI: 0.8923–1.8357), but no difference was detected (*P* > 0.05). RB and XPS required 54.9 ± 17.9 and 22.3 ± 9.3 seconds to perform retreatment procedures, respectively (*P* < 0.05).

The endodontic sealer was evident in all the samples. A larger number of gutta-percha-free samples were found in the XPS group than in the RB group (8 versus 2) (*P*=0.02). In this study, none of the files fractured during the retreatment procedure.

## 4. Discussion

The current in vitro study investigated the debris extrusion for both RB and XPS systems in retreatment cases. Several studies showed improved performance of RB and XPS, compared to other NiTi systems, in many aspects, such as debris extrusion [12, 14, 21], shaping ability [22, 23], removal of obturating material [3, 4, 12, 14], and cyclic fatigue resistance [20, 24]. In our study, compared to RB, XPS displayed a trend of less debris extrusion. The XPS group extruded less debris; however, it was not significant. Alves et al. [9] studied the volume of debris extrusion in initial treatment cases by using microcomputed tomography and found that both systems introduced a similar volume of debris beyond the apex. Another study on initial treatment cases reported that XPS had significantly less debris extrusion, compared to RB, WaveOne Gold, and HyFlex EDM [16]. Furthermore, Azim et al. [12] reported that XPS extruded the least amount of debris in retreatment cases, compared to WaveOne Gold and HyFlex EDM.

An assessment of the preparation time in this study showed that XPS could perform the retreatment in 60% less time than RB. This is consistent with a previous study that found XPS required shorter retreatment time than the other tested systems [12]. Furthermore, XPS was more efficient in gutta-percha removal, and the difference was statistically significant between the groups. This coincides with other studies [4, 12].

The endodontic sealer was evident in all the samples, while the gutta-percha was successfully removed in 53% and 13% of the XPS and RB samples, respectively. Previous studies demonstrated that no instrumentation technique can render the canal free of obturating materials [5, 25, 26]. This coincides with a recent study that found XPS superior to RB and Reciproc [25]. This performance might be explained by the shape of the XPS file, which allows maximum canal wall contact, leading to a greater percentage of touched walls [25]. It is worth mentioning that no solvent or active irrigating solutions were used in this study to purely investigate the effectiveness of rotary systems to remove the obturating materials [27]. Although a good number of shaping strokes (20–40 strokes) in the canal were performed against the walls, the remaining obturating materials were left. De-Deus et al. [25] showed that XPS, RB, and Reciproc were not able to remove the obturation material completely. Therefore, activation of irrigating solutions during the root canal retreatment has been suggested to enhance the removal of filling materials in oval canals [28]. The effect of different irrigation materials and agitation techniques on debris extrusion should be further assessed.

Many attempts have been made to reduce the debris extrusion in clinical practice. However, the literature shows that debris extrusion by several NiTi rotary endodontic files is inevitable [12, 14, 16]. The debris extrusion might contain debris and microbes which could cause postoperative pain [6–8]. Alves et al. [9] studied intracanal bacterial reduction and bacterial extrusion on contaminated canals with the *Enterococcus faecalis* species. They reported that although the extruded bacteria counts were higher with XPS compared to RB, XPS reduced the intracanal bacterial reduction more significantly. Further studies are needed to substantiate this finding.

Since XPS is manufactured to be used at high temperatures, the intracanal temperature was simulated in this study. However, the absence of the simulated apical pressure of the periodontal ligament might limit this methodology [29].

## 5. Conclusion

Within the limitations of this study, the retreatment procedure appears to extrude debris beyond the apex, regardless of the rotary system used. XPS was more efficient in the removal of gutta-percha and required less preparation time compared to RB.

## Figures and Tables

**Table 1:** Removal of obturating materials and mean (SD) and median (25th–75th percentile) of the weight of apically extruded debris and preparation time in the tested groups.

System	Removal of gutta-percha	Removal of sealer	Weight (in mg)		Preparation time (in seconds)	
			Mean (SD)	Median (25th–75th percentile)	Mean (SD)	Median (25th–75th percentile)
Reciproc Blue	2/15	0/15	1.1079 (0.3758)	1.235 (0.8923–1.8357)	54.9 (17.9)	56 (39.9–80.7)

XP Shaper	8/15	0/15	1.0732 (0.3420)	1.145 (0.8471–1.8122)	22.3 (9.3)	18 (15.6–26.7)

Statistical test	Chi-square		Mann–Whitney test			

*P* value	0.020	1	0.95		<0.001	

## Data Availability

The datasets used to support the findings of this study are available from the corresponding author upon request.
